# Quality not just quantity: how health system strengthening is essential for scale up of quality mental health care

**DOI:** 10.1017/S2045796020000980

**Published:** 2020-11-20

**Authors:** Charlotte Hanlon

**Affiliations:** 1 Health Service and Population Research Department, Centre for Global Mental Health, Institute of Psychiatry, Psychology and Neuroscience, King's College London, London, UK; 2 Department of Psychiatry, Addis Ababa University, College of Health Sciences, School of Medicine, WHO Collaborating Centre for Mental Health Research and Capacity-Building, Addis Ababa, Ethiopia; 3 Centre for Innovative Drug Development and Therapeutic Trials for Africa (CDT-Africa), College of Health Sciences, Addis Ababa University, Addis Ababa, Ethiopia

In a recent editorial (Petersen *et al*., 2020), the challenge to successful scale-up mental health care is made clear: how do we strengthen health systems to sustainably deliver high-quality mental health care? The two editorials in this series (Mayston *et al*., 2020; Petersen *et al*., 2020) take up the challenge in complementary ways, both focusing on opportunities that must be seized to ensure that mental health is an integral part of new and emerging initiatives in global health: (1) global calls for a shift to people-centred health systems (Agyepong *et al*., 2017), (2) policy and health system transformation to deliver effective care for people with chronic conditions (Epping-Jordan *et al*., 2004), and (3) ever more accessible technological solutions to develop information systems that will provide the foundation to improve care.

There has been increasing recognition of the need for a health system-based approach to mental health care scale-up. At the time when global mental health first burst onto the global health scene, packages of care based on evidence-based interventions were identified for key, ‘priority’ mental health conditions. This led to a focus on identifying the strategies needed to implement these packages of care in routine care settings. One of the first research-based efforts to do this was the Programme for Improving Mental health carE (PRIME) (Lund *et al*., 2012). In PRIME, there was participatory development, implementation and scale-up of district level, integrated mental health care plans, based on evidence-based packages of care. Some of the key learning from PRIME was around the importance of health system strengthening to ensure the successful implementation of mental health care. In the complementary Emerald project, the health system bottlenecks to high-quality task-shared mental health care were identified leading to recommendations for various health system strengthening interventions (Petersen *et al*., 2019). In South Africa, Petersen *et al*. have taken this a step further, working closely with local government to introduce system innovations that are needed to embed quality mental health care at scale. How does this accumulating evidence and experience link in with the global agenda for high-quality health systems?

In a recent Lancet Commission on high-quality health systems needed to achieve the sustainable development goals (Kruk *et al*., 2018), an ‘epidemic’ of poor-quality health care in low- and middle-income countries was identified. The consequences of this poor quality of health care are immense, resulting in a higher burden of mortality than that attributed to lack of access to care, as well as leading to morbidity and loss of trust in the health care system. For mental health conditions, it is the low access to mental health care (the treatment gap) that continues to dominate the picture and, therefore, research efforts. An estimated 5/100 000 deaths are expected to be amenable to improvement in the quality of mental health care, but non-utilisation of care rather than poor-quality care accounts for more than 80% of deaths that might be averted through health systems. Even so, it is likely that the low quality of mental health, including experience of disrespectful or abusive care, is an important factor in the low uptake of mental health care. Furthermore, advocacy for expanded access to mental health care comes with an ethical imperative to also ensure that care is of high quality (competent care and systems, positive user experience) and can deliver the ‘quality impacts’ of improved health, economic benefits of health care and confidence in the health system (Kruk *et al*., 2018).

In relation to the four values underpinning high-quality health systems (‘for the people’, equitable, resilient and efficient) (Kruk *et al*., 2018), Petersen *et al*. focus on health systems being ‘for the people’. People-centred mental health care is at the heart of the Innovative Care for Chronic Conditions Framework, which underpins the service delivery structure being scaled up in South Africa and has been adapted for other settings in Africa (Epping-Jordan *et al*., 2004). Key system barriers to shifting to a people-centred health system include the historical legacy of services orientated to acute episodes of infectious disease, vertical programming, paternalistic clinician–patient relationships and cultures of disrespectful care underpinned by over-burdened, inadequately supported health care providers with high levels of burnout (Petersen *et al*., 2019). The lack of people-centred care is an important factor contributing to the low detection and treatment of co-morbid mental health conditions in general healthcare settings. In South Africa, this is being tackled through a multi-level health system strengthening innovations. The Practical Approach to Care Kit provides evidence-based decision-support to primary healthcare workers in a ‘one-stop-shop’ (combining guidelines across all common conditions presenting to primary care), integrates care for multimorbid conditions and defines the health worker's scope of practice (Fairall *et al*., 2020). The linked clinical communications skills toolkit seeks to care for the wellbeing of health workers and equip them for a new way of working which is holistic (responding to multimorbidity, psychosocial needs and social determinants of ill-health) and grounded in the idea of service users as partners in care. Facilitative policies, plans and structures have also been implemented, including training and supervision cascades, job descriptions, aligned performance monitoring targets, referral pathways and pre-service training. Focusing on people-centred care is essential for effective scale-up of quality mental health care integrated into general health care settings while strengthening the health system more broadly for the benefit of all service users.

Data that can be used for accountability of the system or to promote action to improve care are foundational for a high-quality health system (Kruk *et al*., 2018). In the editorial by Petersen *et al*. (Petersen *et al*., 2020), continuous quality improvement (CQI) of mental health care, driven by timely and relevant data on system performance, has been incorporated into scale-up through CQI leads who mentor the process on an ongoing basis. Successful feedback of learning from CQI to stakeholders has already led to actions that can strengthen the health system, for example, stimulating political will for pre-service training in mental health care. Mayston *et al*. illustrate how accurate, relevant and timely information is necessary to achieve several elements of the Innovative Care for Chronic Conditions Framework: at the individual level for proactive, person-centred care, at the facility level for supervision, performance monitoring and case-load management, at the macro level for governance and accountability, and for fuelling CQI and co-learning to strengthen health systems in the longer term (Mayston *et al*., 2020).

As Mayston *et al*. describe, tracking service user progress along the care continuum, from detection, initiation on an appropriate care pathway, retention in care and treatment to target (defined in terms that are meaningful to the individual), is vital for high-quality care but is not easily achieved using paper-based approaches. Technology, for example, in the form of mHealth applications, has great potential to solve this problem through the automated aggregation and display of information in real time to inform local action. Although information may be disease-specific, for example, treatment to the target of depression may be framed in terms of reduction of depressive symptoms below a pre-specified threshold, this information can also provide a window to the wider performance of the health system. For example, low levels of detection of co-morbid conditions across different chronic diseases (e.g. HIV, TB, hypertension) can signal failures of integrated care. Low levels of initiation on evidence-based pathways can indicate problems with competent care. Failure to treat to target raises questions about adequacy of supervision and mentoring of staff.

A major impediment to achieving high-quality mental health systems is the lack of demand for quality care from people with mental health conditions and the wider community. Stigma (enacted and self-stigma), discrimination and abuse towards people with mental health conditions are potent obstacles to their involvement in strengthening health systems to deliver high-quality care. In the eyes of the community, specialist mental health services are often equated with coercion and abuse, undermining confidence in the care provided and fuelling a lack of trust in the system. Marginalisation and low levels of empowerment of mental health service users are associated with low levels of awareness of their rights and low expectations of care. Although there is limited published evidence of mental health service user involvement in health system strengthening, initiatives are underway. Empowerment approaches are being used to equip service users with the skills, confidence and support they need to express their experiences and preferences to health workers and healthcare planners, with a view to effecting improvements in care (Kohrt *et al*., 2020). Participatory action research is being used in rural Ethiopia to inform people with mental health conditions about their right to quality care and to mobilise people for action (Abayneh *et al*., 2020). In the ASSET project (health system strengthening in sub-Saharan Africa: www.healthasset.org), mental health service users will join CQI activities with health workers in learning health systems with the goal of improving care processes (including user experience) and the impact of care. Empowered and engaged mental health service users hold perhaps the biggest promise for driving forward a quality revolution for mental health care globally.

## References

[ref1] Abayneh S, Lempp H, Alem A, Kohrt B, Fekadu A and Hanlon C (2020) Developing a theory of change model of service user and caregiver involvement in mental health system strengthening in primary health care in rural Ethiopia. International Journal of Mental Health Systems 14, 51. 32760440 10.1186/s13033-020-00383-6 PMC7379363

[ref2] Agyepong IA, Sewankambo N, Binagwaho A, Coll-Seck AM, Corrah T, Ezeh A, Fekadu A, Kilonzo N, Lamptey P, Masiye F, Mayosi B, Mboup S, Muyembe J-J, Pate M, Sidibe M, Simons B, Tlou S, Gheorghe A, Legido-Quigley H, McManus J, Ng E, O'Leary M, Enoch J, Kassebaum N and Piot P (2017) The path to longer and healthier lives for all Africans by 2030: the Lancet Commission on the future of health in sub-Saharan Africa. The Lancet 390, 2803–2859. 10.1016/S0140-6736(17)31509-X 28917958

[ref3] Epping-Jordan JE, Pruitt SD, Bengoa R and Wagner EH (2004) Improving the quality of health care for chronic conditions. Quality & Safety in Health Care 13, 299–305. 15289634 10.1136/qshc.2004.010744 PMC1743863

[ref4] Fairall L, Cornick R and Bateman E (2020) Empowering frontline providers to deliver universal primary healthcare using the practical approach to care kit. BMJ Global Health 3, ek4451rep. 10.1136/bmjgh-2018-k4451rep PMC6266967 30538824

[ref5] Kohrt BA, Turner EL, Rai S, Bhardwaj A, Sikkema KJ, Adelekun A, Dhakal M, Luitel NP, Lund C, Patel V and Jordans MJD (2020) Reducing mental illness stigma in healthcare settings: proof of concept for a social contact intervention to address what matters most for primary care providers. Social Science & Medicine 250, 112852. 32135459 10.1016/j.socscimed.2020.112852 PMC7429294

[ref6] Kruk ME, Gage AD, Arsenault C, Jordan K, Leslie HH, Roder-DeWan S, Adeyi O, Barker P, Daelmans B, Doubova SV, English M, Elorrio EG, Guanais F, Gureje O, Hirschhorn LR, Jiang L, Kelley E, Lemango ET, Liljestrand J, Malata A, Marchant T, Matsoso MP, Meara JG, Mohanan M, Ndiaye Y, Norheim OF, Reddy KS, Rowe AK, Salomon JA, Thapa G, Twum-Danso NAY and Pate M (2018) High-quality health systems in the sustainable development goals era: time for a revolution. The Lancet Global Health 6, e1196–e1252. 30196093 10.1016/S2214-109X(18)30386-3 PMC7734391

[ref7] Lund C, Tomlinson T, De Silva M, Fekadu A, Shidhaye R, Jordans M, Petersen I, Bhana A, Kigozi F, Prince M, Thornicroft G, Hanlon C, Kakuma R, McDaid D, Saxena S, Chisholm D, Raja S, Kippen-Wood S, Honikman S, Fairall L and Patel V (2012) PRIME: a programme to reduce the treatment gap for mental disorders in five low- and middle-income countries. PLoS Medicine 9, e1001359. 23300387 10.1371/journal.pmed.1001359 PMC3531506

[ref8] Mayston R, Ebhohimen K and Jacob K (2020) Measuring what matters – information systems for management of chronic disease in primary healthcare settings in low and middle-income countries: challenges and opportunities. Epidemiology and Psychiatric Sciences 29, e127. 32389151 10.1017/S204579602000030X PMC7232121

[ref9] Petersen I, van Rensburg A, Kigozi F, Semrau M, Hanlon C, Abdulmalik J, Kola L, Fekadu A, Gureje O, Gurung D, Jordans M, Mntambo N, Mugisha J, Muke S, Petrus R, Shidhaye R, Ssebunnya J, Tekola B, Upadhaya N, Patel V, Lund C and Thornicroft G (2019) Scaling up integrated primary mental health in six low- and middle-income countries: obstacles, synergies and implications for systems reform. BJPsych Open 5, e69. 31530322 10.1192/bjo.2019.7 PMC6688466

[ref10] Petersen I, van Rensburg A, Gigaba SG, Luvuno ZBP and Fairall LR (2020). Health systems strengthening to optimise scale-up in global mental health in low- and middle-income countries: lessons from the frontlines. A re-appraisal. Epidemiology and Psychiatric Sciences 29, e135, 131–135. 10.1017/S2045796020000475 PMC7303791 32536359

